# Detector Response to D-D Neutrons and Stability Measurements with 4H Silicon Carbide Detectors

**DOI:** 10.3390/ma14030568

**Published:** 2021-01-26

**Authors:** Matteo Hakeem Kushoro, Marica Rebai, Marco Tardocchi, Carmen Altana, Carlo Cazzaniga, Eliana De Marchi, Francesco La Via, Laura Meda, Alessandro Meli, Miriam Parisi, Enrico Perelli Cippo, Mario Pillon, Antonio Trotta, Salvo Tudisco, Giuseppe Gorini

**Affiliations:** 1 Dipartimento di Fisica “G. Occhialini”, Università di Milano-Bicocca, 20126 Milano, Italy; giuseppe.gorini@unimib.it; 2 Istituto per la Scienza e Tecnologia dei Plasmi, Consiglio Nazionale delle Ricerche, 20126 Milano, Italy; marica.rebai@istp.cnr.it (M.R.); marco.tardocchi@istp.cnr.it (M.T.); enrico.perellicippo@istp.cnr.it (E.P.C.); 3 Istituto Nazionale di Fisica Nucleare (INFN), Laboratori Nazionali del Sud (LNS), Via S. Sofia 62, 95123 Catania, Italy; altana@lns.infn.it (C.A.); tudisco@lns.infn.it (S.T.); 4 ISIS Facility, UKRI-STFC, Rutherford Appleton Laboratory, Didcot OX110 QX, UK; carlo.cazzaniga@stfc.ac.uk; 5 ENI S.p.A. CENTR—Renewable Energy & Environmental R&D, 28100 Novara, Italy; Eliana.DeMarchi@eni.com (E.D.M.); laura.meda@eni.com (L.M.); miriam.parisi@eni.com (M.P.); Antonio.Trotta@eni.com (A.T.); 6 Institute for Microelectronics and Microsystems, Consiglio Nazionale delle Ricerche VIII Strada, 5, 95121 Catania, Italy; francesco.lavia@imm.cnr.it (F.L.V.); Alessandro.Meli@imm.cnr.it (A.M.); 7 Associazione EURATOM-ENEA sulla Fusione ENEA C.R. Frascati, Via E. Fermi, 45, 00044 Frascati, Italy; mario.pillon@enea.it

**Keywords:** silicon carbide, fast neutron detection, tokamak

## Abstract

The use of wide-band-gap solid-state neutron detectors is expanding in environments where a compact size and high radiation hardness are needed, such as spallation neutron sources and next-generation fusion machines. Silicon carbide is a very promising material for use as a neutron detector in these fields because of its high resistance to radiation, fast response time, stability and good energy resolution. In this paper, measurements were performed with neutrons from the ISIS spallation source with two different silicon carbide detectors together with stability measurements performed in a laboratory under alpha-particle irradiation for one week. Some consideration to the impact of the casing of the detector on the detector’s counting rate is given. In addition, the detector response to Deuterium-Deuterium (D-D) fusion neutrons is described by comparing neutron measurements at the Frascati Neutron Generator with a GEANT4 simulation. The good stability measurements and the assessment of the detector response function indicate that such a detector can be used as both a neutron counter and spectrometer for 2–4 MeV neutrons. Furthermore, the absence of polarization effects during neutron and alpha irradiation makes silicon carbide an interesting alternative to diamond detectors for fast neutron detection.

## 1. Introduction

Solid State Detectors (SSDs) represent a recent option for neutron detection in high-flux applications, since they combine a good pulse height energy resolution and fast response time while having compact dimensions [1,2]. The SSD scene is currently dominated by diamond detectors, which, for instance, are currently installed at the JET tokamak [3] as neutron spectrometers [2,4] and at the ChipIr beamline at ISIS [5] as beam monitors [6,7]. However, the development of large high-power tokamaks (such as ITER [8]) requires neutron detectors to be installed closer to the plasma and, therefore, to be able to sustain the high temperature and neutron flux of such an environment. This is driving interest in new and more neutron-resilient SSDs, such as silicon carbide detectors.

Silicon carbide detectors (SiC) are a type of SSD whose active volume is made from silicon carbide, a crystalline material known for its resilience and high radiation hardness since the late 1950s [9]. SiC can withstand high temperatures [10], radiation [11] and neutron fluxes [12]; furthermore, in recent years, new manufacturing techniques have allowed the production of SiC detectors with fewer defects and with a wider range of geometries [13]. The detector responses of these new SiC detectors were characterized in the past with 14 MeV Deuterium-Tritium (D-T) neutrons [14] and over a wide range of fast neutron energies [15], showing energy resolutions and efficiencies comparable to those of the diamond detectors.

The objective of this paper was to expand the knowledge on SiC for use in next-generation tokamaks as a neutron counter and spectrometer. To achieve this, the stability of two models of SiC under long-term neutron and α-particle irradiation was experimentally investigated and compared to the stability limits of state-of-art diamond detectors ([16,17]) in order to find the best candidate for measuring prolonged irradiation. Some consideration is given to the effect of the casing on the detector’s counting rate. Finally, the characterization conducted in [14] was expanded to D-D neutrons, which are the second most important neutrons emitted from fusion plasma besides D-T neutrons, in order to assert SiC’s ability to act as a neutron spectrometer in the 2 to 4 MeV energy range.

## 2. Detectors and Front-End Electronics

SiC detectors are made of a thin semi-conductive lattice that acts both as a converter and as an active volume. The lattice is built as a p–n junction and behaves like a diode [18]. The interaction of a fast neutron with a silicon (Si) or carbon (C) nucleus induces the generation of charged particles via nuclear reactions or via Si or C recoil ions through scattering collision. These charged particles and ions, in turn, ionize the p–n junction and generate a number of ion–electron pairs proportional to the energy deposited by the charged particles ($E_{d}$). The pairs abruptly decrease the resistivity of the p–n junction, causing a current signal that is used as a detection mechanism. There is a linear proportionality between $E_{d}$ and the signal amplitude, allowing for the use of SSDs as spectrometers.

SSDs can also be used to detect heavy charged particles (such as alpha particles or protons), which directly generate ion–electron pairs in the lattice via ionization/excitation processes without the need to be converted. The detector is also sensitive to γ-rays, although the sensitivity to them is much lower than the one to neutrons since carbon and silicon have much smaller cross sections for γ-rays than for neutrons and the detector has a small thickness. This is actually a desirable feature for a neutron detector, as γ-rays constitute the most intense source of background in almost all neutron facilities.

Two SiC detectors were used in this paper, both designed and manufactured at the Institute for Microelectronics and Microsystems of the Consiglio Nazionale delle Ricerche (CNR), based in Catania (Italy). The detectors’ active volumes were realized by growing 4H-silicon carbide epitaxial layers by means of Chemical Vapor Deposition (CVD). p–n junction doping was achieved by adding N₂ and Al_2_(CH_3_)_6_ (trimethylaluminum) to the silicon and carbon gaseous precursors in order to obtain nitrogen and aluminum impurities. The wafers were then treated with several photolithographic steps, among which were Inductive Coupled Plasma (ICP) etching for the definition of the detector area; lithography to construct the edge structures; the deposition of an isolation oxide, with subsequent annealing, in order to produce good electrical contact on the p+ region; and titanium/nickel/gold deposition in order to form ohmic contact. More details of the process can be found in [13,19].

**Figure 1:**
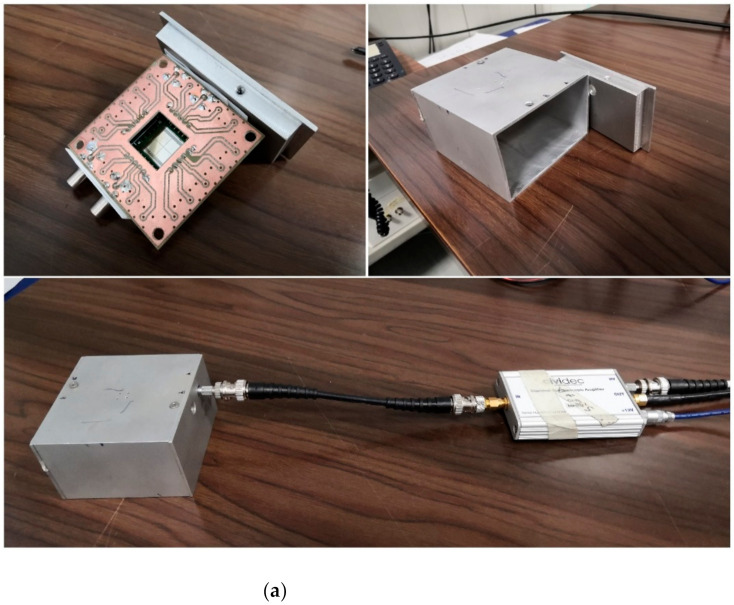

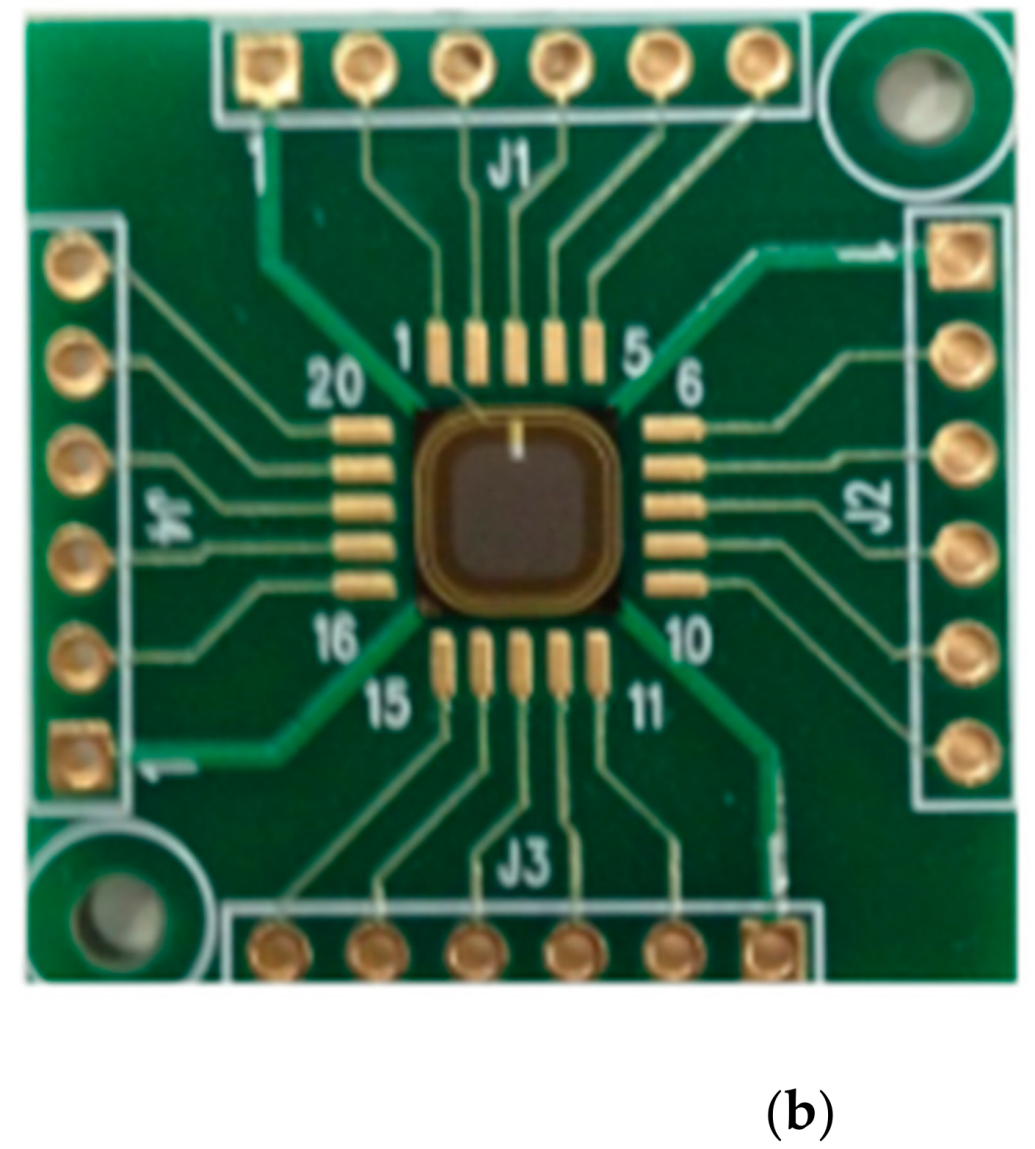
(**a**) The 100 μm silicon carbide detector used in this paper. The four independent pixels and the chip electronics are visible in the center of the top-left figure. The aluminum casing in which it was contained is shown in the top right. The detector assembled and connected to the spectroscopic preamplifier is shown in the bottom figure. (**b**) The chip and the single active volume of the 10 μm silicon carbide detector used in this paper.

The first detector used was made with a single active volume 5 mm × 5 mm wide and 10 µm thick (Figure 1b). The second was made of a 2 × 2 matrix of independent 5 mm × 5 mm-wide and 100 µm-thick active volumes, all grown on the same substrate (Figure 1a). Only one of the four pixels was actively used for the detection.

The active volume of both detectors, which will be referred to as SiC10 and SiC100 from now on, was a 0.3 µm-thick layer with a $10^{19}{\;\mathrm{cm}}^{-3}$ doping concentration of Al, matched to a 10 or 100 µm p-layer with a $N_{2}$ concentration between $8\times 10^{13}\;$ and $10^{14}{\;\mathrm{cm}}^{-3}$. The inactive substrate of the detectors was 100 µm thick in the case of SiC10 and 350 µm thick in the case of SiC100. Each of the two was encased in an aluminum box between 3 and 7 mm thick, in order to shield the detector from environmental electromagnetic radiation and from dust.

The output signal of the detector was transmitted through 50 Ω coaxial cables and preamplified with a customized charge preamplifier (model CIVIDEC CX-L, manufactured by CIVIDEC Instrumentation GmbH, Wien, Austria), which shaped the signal into a pseudo-Gaussian shape. The CX-L preamplifier features the best energy resolution and signal-to-noise ratio and has a typical shaping time of ≃280 ns. Alternatively, in some measurements, a current amplifier (model CIVIDEC C2) was used. Being a current amplifier, the C2 does not shape the detector signal, which makes it more suited for applications where high fluxes are an issue since it has a much faster response (≃15 ns) [20].

The preamplifiers also convey the bias voltage to the detector. Negative bias voltages of −200 V for the SiC100 and −50 V for the SiC10 were chosen in order to deplete the active volumes of both detectors [14], without incurring any discharges [19]. With such bias voltages, the drift times were of the order of nanoseconds [21]; therefore, with such fast signals, any limitations in the detection rate were imposed by the analyzing chain.

The signal was then read by a 14-bit/500 MHz sampling rate digitizer (model DT5730, manufactured by CAEN, Viareggio, Italy), which allowed storing the data on the PC and recording the timestamp and the pulse integral for each event. The pulse height spectrum was then reconstructed from the stored events.

## 3. Irradiation Facilities

### 3.1. ISIS (ChipIr and ROTAX)

The ISIS Neutron and Muon Source is a spallation source at the UKRI-SFTC Rutherford Appleton Laboratory in Didcot (UK). Neutrons are produced through spallation by a pulsed 800 MeV Proton Synchrotron (PS) beam impinging onto two different tungsten targets (Target Stations 1 and 2). The PS operates in 50 Hz pulsed mode. Every pulse is made by two 70 n- wide proton bunches separated by a time interval of 322 ns. After the spallation process, neutrons, which are emitted in all directions, are firstly reflected by a beryllium assembly, moderated and then collimated in different beam lines, resulting in white neutron spectra ranging from fast (up to 800 MeV) to cold neutrons (<1 meV). Neutrons travel to the various experimental areas, where their energy $E_{n}$ can be measured by means of the time-of-flight technique.

The data for this paper were collected in two different beamlines: ROTAX and ChipIr. The first one is a beamline of the ISIS Target Station 1. It features a 95 K methane moderator, a neutron flight path of 15.5 m, and a thermal neutron flux at the sample position of $\phi_{n_{\mathrm{th}}}≃3\times 10^{6}\frac{n}{{\mathrm{cm}}^{2}\cdot s}$ [22], and it is dedicated to the characterization of single-crystal samples, detectors and equipment. ROTAX features a residual fast neutron flux ($E_{n}>10$ MeV) of about $\phi_{n_{\mathrm{fast}}}≃10^{4}\frac{n}{{\mathrm{cm}}^{2}\cdot s}$ ChipIr, on the other hand, is a beamline of the ISIS Target Station 2 that has a direct line of sight to the target (not the moderator), and thus, it features a fast neutron spectrum. It is dedicated to the irradiation of microelectronics with a high-energy neutron flux. ChipIr features an atmosphere-like neutron spectrum with a fast neutron flux of $\phi_{n_{\mathrm{fast}}}≃5\times 10^{6}\frac{n}{{\mathrm{cm}}^{2}\cdot s}$ for $E_{n}>10$ MeV neutrons. It has a neutron flight path of 10.5 m. A residual thermal flux is also present: ($\phi_{n_{\mathrm{th}}}≃10^{5}\frac{n}{{\mathrm{cm}}^{2}\cdot s}$) [23,24].

### 3.2. FNG

The Frascati Neutron Generator (FNG) is a neutron source based at the ENEA Laboratories in Frascati, Italy. It was specifically built as a research tool for thermonuclear controlled fusion. Neutrons are produced by a 260 keV deuteron beam impinging on a deuterated target via the nuclear reaction $D{\left(D,n\right)}^{3}He$. The reaction is exothermic: the positive q-value $Q_{v}$ = +3.27 MeV is split between the two reaction products. The neutron energy depends on the emission angle and ranges from a maximum of $E_{n}$ = 3.15 MeV, for a neutron emitted with a ϕ = 0° angle (forward direction), to a minimum of $E_{n}$ = 2.0 MeV, for a neutron emitted with a ϕ = 180° angle. By placing the detector at a known angle ϕ, it is therefore possible to expose it to a quasi-monochromatic neutron beam (if the solid-angle contribution and the beam-energy broadening due to the Doppler effect are neglected).

### 3.3. Alpha Irradiation

Alpha (α) irradiation was carried out at the Istituto per la Scienza e Tecnologia dei Plasmi (ISTP) laboratories in Milano, Italy. The source used was an Americium-241 electrodeposited radioactive source, which emits monochromatic 5.49 MeV α particles along with 60 keV X-rays. Since the half-life of ^241^Am is 432 years, the source was assumed to have a constant emission during the period of the measurement.

The source was placed at 16.4 mm from the SiC10 surface and at 25 mm from the SiC100. For this reason, the energy of the α impinging on the detector surface was slowed down to 4.7 and 4.3 MeV, respectively, by air [25]. The holes in the casing that were purposely made for the α particles to reach the detector’s surface also granted collimation, limiting the maximum drift from the shortest path to 0.23 mm for the SiC10 and 0.14 mm for the SiC100: this limited the broadening of the energy spectrum of the α particles to 19 and 12 keV, respectively, which corresponded to 0.4% and 0.28% of the relative energies. As the energy resolution of the detector is expected to be one order of magnitude higher [14,15], the α particles can be safely assumed to be monochromatic for the purposes of this paper.

## 4. Stability Measurements

With “detector stability”, we usually refer to the capability of a detector to operate for long irradiation times without altering its response. There are several factors that can alter the response of an SSD, the main one being that the prolonged exposure to ionizing radiation results in free charges trapped inside the lattice that alter the drift electric field. Diamond detectors have shown to be subject to this effect [17]. This is an issue even if the restoration of the initial condition can be achieved by an inversion in the polarity of the detector [16] since it is desirable for a detector in a fusion or spallation environment to be able to operate continuously.

Diamond detectors’ stability was measured in the past [16,17], and they were proven to be stable up to $7\times 10^{3}\frac{\alpha }{{\mathrm{mm}}^{2}}$, after which the count rate dropped significantly. The same was tested with neutrons, showing a 20% decrease in the number of counts after a neutron fluence of $1.2\times 10^{6}\frac{n}{{\mathrm{mm}}^{2}}$ The measurements described in this paper emulated those tests by using the two SiC detectors and exposing them to α particles and neutrons in the facilities previously described.

### 4.1. Results for Stability with Alpha Particles

The signal of the α particles produced by the ^241^Am source was collected by both the SiC10 and the SiC100 detectors. The preamplifier used in these measurements was the CX-L model mentioned earlier. The exposure lasted for 55 and 170 h, respectively, which provided a total detector irradiation of $7.9\times 10^{6}$ and $9.0\times 10^{6}\frac{\alpha }{{\mathrm{mm}}^{2}}$.

**Figure 2:**
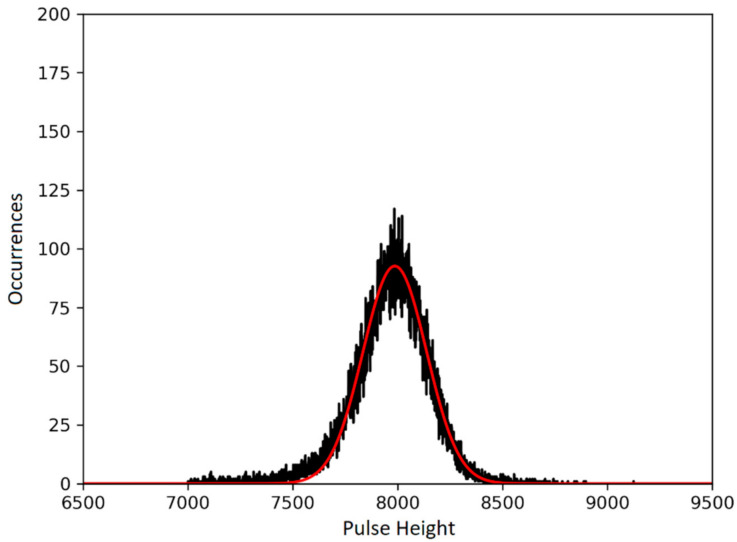
Pulse height distribution for a 300 s exposure of the SiC10 to 4.7 MeV α particles (black line), fitted with a Gaussian function (red line), obtaining a mean pulse height of 7986.34. FWHM is 386.94, which provided an energy resolution of 4.85%.

Both the time and the pulse height of the events were recorded. A threshold of 200 keV was applied, in order to not consider the X-rays produced by the ^241^Am source and other background noise. The events were then grouped in 300 s time intervals. For every group, a histogram of the events’ pulse heights was made (see Figure 2). The histograms were fitted with a Gaussian function, whose central value $x_{0}$ was taken as the mean pulse height for the ensemble. The Full Width at Half Maximum (FWHM) was also obtained through the relation FWHM = $\;2\sigma \times \sqrt{2\times \ln\left(2\right)}$, where σ is the standard deviation of the Gaussian fit. The energy resolution of the detector was then computed as the ratio between the FWHM and the alpha energy, with the assumption that the incident α-particle spectrum was monochromatic (see Section 3.3).

**Figure 3:**
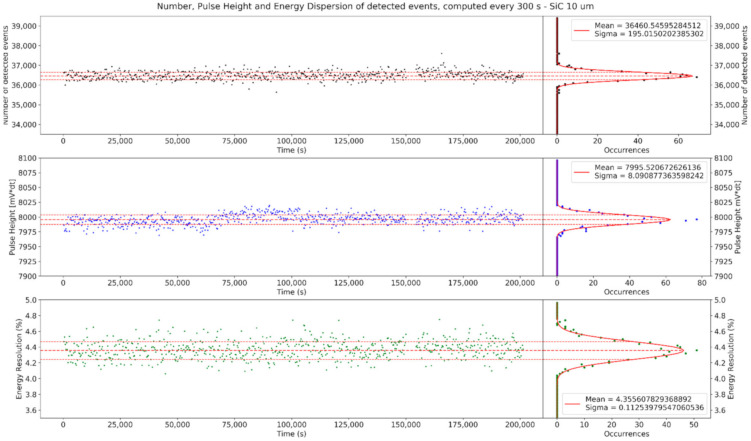

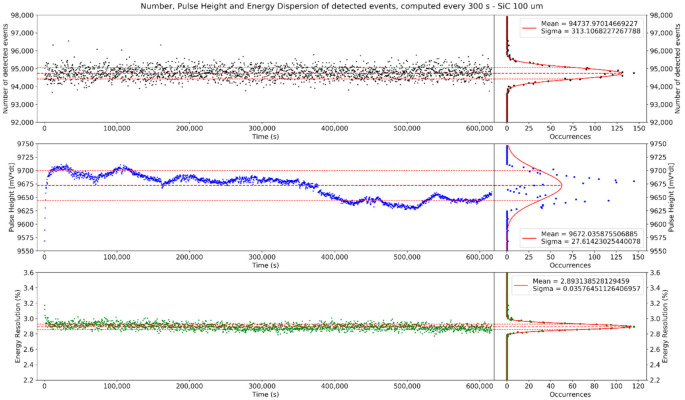
Time evolution of the number of counts (black), the mean pulse height (blue) and the energy resolution (green) for the 10 µm SiC (**top**) and the 100 µm SiC (**bottom**) under irradiation with quasi-monochromatic 4.7 and 4.3 MeV αs, respectively. SiC10 and SiC100 alpha irradiation lasted for 55 and 170 h, respectively. Each of the points corresponds to a 300 s ensemble. On the right, the dispersion of data is projected on the y-axis, and the mean value of the three quantities was computed, along with its standard deviation.

The number of counts, the mean pulse height and the energy resolution were calculated for every 300 s subset and are plotted in Figure 3 as functions of time. The mean values and dispersions obtained by the fits are then collected in Table 1. Neither the number of counts nor the FWHM had a drift over the multiple-day-long acquisition. The energy resolutions obtained were 4.3% for the SiC10 and 2.9% for the SiC100, the latter confirming the resolution obtained in [15] and in [16]. The overtime trend of the energy resolution did not have any significant drift over the multiple-day exposure and shows only a small Gaussian-shaped dispersion (2–3% of the energy resolution value), which can be interpreted as a statistical error. This proves the stability of the energy resolution over the analysis period. The same considerations may apply to the number of counts, which is linked to the detector’s efficiency. For both the SiC10 and the SiC100, the σ of the dispersion around the number of counts perfectly fits the Poisson uncertainty for the number of counts, μ, given by $\sqrt{µ}$, as shown in the first panel of Table 1.

The same consideration may not be applied to the response function, since the mean value of the pulse height has a slight periodic oscillation and a drift that is dependent on time and, thus, cannot be interpreted as a random error due to statistics. It is worth noting that the change in the response function of the two detectors does not have any correlation with the number of counts or the energy resolution. While the cause of this periodic drift is still unclear, the lack of correlation with the other two parameters suggests that the oscillation could be due to a variability in the energy of the ^241^$Am_{}$ α particles impinging on the detector rather than a change in the detector response. Since the period of the oscillation is ≃85,000 s (=23.61 h), it has been hypothesized that the cause is the variability of the air-stopping power caused by a change in the pressure of the air, presumably due to the night–day cycle. Indeed, the lab is estimated to have had a thermal excursion between 27 and 30 °C, which would have caused a 1% excursion in the air density (1.176 $\frac{\mathrm{kg}}{m^{3}}$ at 27 °C and 1.164 $\frac{\mathrm{kg}}{m^{3}}$ at 30 °C, if air humidity is neglected). Since air-stopping power scales linearly with density [25], this reflects a 1% change in the energy of the impinging alpha particle, which is compatible with the 0.87% difference between the maximum and minimum values for the pulse height in the second panel of Table 1.

### 4.2. Results for Stability with Neutrons

The detector stability with neutrons was tested at ChipIr with the SiC10 and at ROTAX with the SiC100. The detectors were exposed to the ISIS pulsed neutron flux, and the pulse height of the events was measured in a 2 µs time window synchronized with the start signal from the PS beam. The window allowed limiting the detection to neutrons with $E_{n}$ > 0.5 MeV, as obtained from the non-relativistic relation:

$$E_{n}\left[MeV\right]=\frac{m_{n}\times d{\left[m\right]}^{2}}{2\times 10^{-18}\times \;ToF\left[ns\right]}$$

where $m_{n}$ is the neutron mass in Kg, *d* is the flight path of the neutron (10.5 m at ChipIr; 15.5 m at ROTAX) and ToF is the time of flight, calculated as the time difference between the time of generation of the neutrons and the time of arrival of the event. The former was obtained from the time of the first detected events, under the assumption that those were caused by γ-rays travelling at the speed of light *c*. The preamplifier used was the CIVIDEC C2 for both the ROTAX and ChipIr experiments. The exposure of the SiC10 at ChipIr lasted for 48 h, with an estimated total neutron flux of $9.06\times 10^{8}\frac{n}{{\mathrm{mm}}^{2}}$ (above 0.5 MeV), while the exposure of the SiC100 lasted for 96 h, with an estimated total neutron flux of $2.59\times 10^{8}\frac{n}{{\mathrm{mm}}^{2}}$ (above 0.5 MeV). It must be noted that these fluxes correspond only to the fast neutrons that were detected inside the detection windows: both detectors were also exposed to an undetermined number of slower neutrons, as no shutter was used in order to shield the detection areas from them.

**Figure 4:**
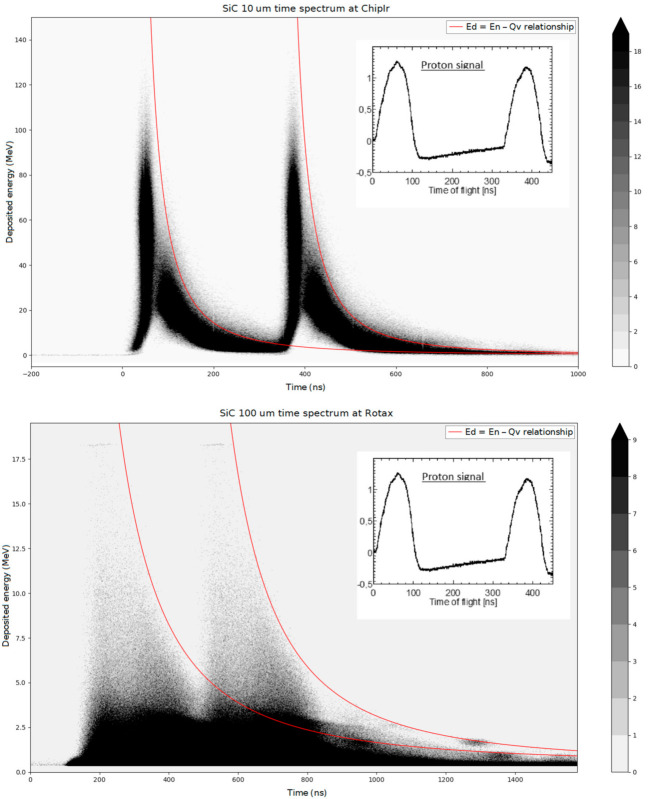
Event density plot (in the time of flight (ToF) vs. $E_{d}$ space) of 100 μm SiC on ROTAX (top) and of 10 μm SiC on ChipIr (bottom). The two-bunch Proton Synchrotron (PS) spectrum is showcased on the right. The red lines represent the relationship between ToF and $E_{n}$ and are separated by 322 ns, mirroring the PS spectrum.

The events recorded both at ROTAX and at ChipIr are shown in Figure 4 in the form of two event density plots, in analogy to the plots of [6,14,15,26]. The data are grouped into two bunches, reflecting the time structure of the proton beam. The red lines represent the relationship between $E_{n}$ and ToF and are separated by 322 ns, which mirrors the time difference between the two PS proton bunches. All the events detected fall below those lines, validating the data.

**Figure 5:**
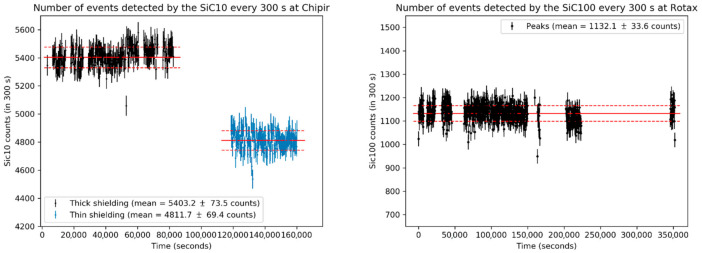
The numbers of events detected every 300 s by the SiC10 on ChipIr (**left**) and by the SiC100 on ROTAX (**right**) are plotted as functions of time for 44 and 98 h-long exposures, respectively. Data from the SiC10 are divided into the data taken with thick aluminum shielding (in black) and those taken with thin shielding (in blue). Red lines represent the count rates, while dashed red lines are the statistical errors for the numbers of counts.

Stability was tested for both the ChipIr and ROTAX data by tracking the evolution over time of the number of events detected in every 300-second ensemble, in analogy to the α-irradiation. Contrary to for the α-irradiation, it was not possible to test the evolution of the response function and of the energy resolution, because the flight paths of both Rotax and ChipIr are too short and the proton beam temporal structure too wide to be able to obtain, for fast neutrons, $E_{n}$ from the ToF with a sufficient energy resolution. The number of incident neutrons was assumed to be constant, since the number of PS protons fired on the target was proven to be stable with an error of ±1% for the duration of operation. The results are shown in Figure 5.

The number of counts was fitted with a constant value µ (full red line) corresponding to the mean value of counts per 300 s. Such a value is $\mu_{\mathrm{SiC}10}=\;$ 5403 $\pm \;74\;\frac{\mathrm{counts}}{300\;s}$ for the SiC10 at ChipIr and $\mu_{\mathrm{SiC}100}=$ 1132 $\pm \;34\frac{\mathrm{counts}}{300\;s}$ for the SiC100 at ROTAX. The error was calculated from the Poisson uncertainty around the number of counts, which is equal to $\pm \sqrt{\mu }$. As the majority of the data fit the value within the error, and no drift over time was observed, the detector was proven stable over the range of fluences tested, which are at least two orders of magnitude higher than the stability fluency ranges for the diamond measured in [17].

A notable exception is given by the subset of the final 8-hour irradiation at ChipIr (highlighted in blue in Figure 5), which features a number of counts of $\mu =\;4812\pm 70\;\frac{\mathrm{counts}}{300\;s}$. Such a number is 11% lower than the mean value for the rest of the data, while the PS current was the same as for all the other irradiations (within the error). It must be noted that the subset was taken after the front part of the 3 mm aluminum casing was removed and replaced with a thin 50 μm aluminum foil. As such, the difference in the number of counts is supposed to be due to the number of neutrons that were converted to protons in the aluminum casing through the reaction ${}^{27}Al\left(n,p\right){}^{27}Mg$; these protons, being charged particles, have an almost 100% chance of being detected by the SiC, thus increasing the number of counts under the same neutron irradiation.

In order to estimate the impact of this phenomenon, we compared the macroscopic cross sections per unit of area of the 3 mm aluminum casing and one of the active volume of the detector. We defined the macroscopic cross section as $\chi =\sigma \times n_{\mathrm{at}}/A$, where σ is the atomic cross section of the nuclear reactions (obtained from the ENDF - Evaluated Nuclear Data File - catalogue [27], averaging over neutrons with $5<E_{n}<20$) and $n_{\mathrm{at}}/A$ is the number of atoms per unit area, derived from the atomic density ρ (using $n_{\mathrm{at}}=\rho \times A\times t$, where t is the thickness of the object). We then compared the macroscopic cross section of the SiC related to all the nuclear reactions induced by the neutron $\chi_{\mathrm{SiC}}$ (derived from the total nuclear cross section σ≃0.4 barn), with the macroscopic cross section of the aluminum casing for the specific ${}^{27}Al\left(n,p\right){}^{27}Mg$ reaction, $\chi_{\mathrm{casing}}$ (σ≃0.03 barn). We obtain:

$$\chi_{\mathrm{SiC}}=9.67\times 10^{22}\;cm^{-3}\times 10\;\mu m\times 0.4\;barn=38.7\times 10^{-6}$$

$$\chi_{\mathrm{casing}}=6.03\times 10^{22}\;cm^{-3}\times 3\;mm\times 0.03\;barn=542.7\times 10^{-6}$$

That is, the number of neutrons that were converted to protons in the thick aluminum shielding were more than thirteen times the neutrons that interacted with the active volume. If we divide the number of protons by the fraction of the solid angle of the detector’s area (assuming the proton emission is isotropic), we obtain the SiC’s macroscopic cross section relative to the converted protons $\chi_{\mathrm{SiC}-p}$:

$$\chi_{\mathrm{SiC}-p}=\;\chi_{\mathrm{casing}}\times \frac{A_{\mathrm{SiC}}}{4\pi \times {\left(15\;mm\right)}^{2}}=4.7\times 10^{-6}$$

where 15 mm is the distance between the casing and the surface of the detector. By assuming that all the protons, being heavy charged particles, are detected, we determine that the estimated ratio of the proton events over the total events is $\frac{\chi_{\mathrm{SiC}-p}}{\chi_{\mathrm{SiC}}\;+\;\chi_{\mathrm{SiC}-p}}=10.8\%$, which is very close to the decrease in the number of counts experienced by the detector after the casing was replaced with a thinner one.

## 5. Neutron Spectroscopy (3.0 MeV)

The peculiar structure of the FNG (described in Section 3.2) allowed for the exposure of the detector to quasi-monochromatic neutrons. The SiC100 was exposed to D-D neutrons to complement the D-T spectroscopy performed in [14] at the same facility. Three angles were chosen (0°, 30° and 50°) in order to characterize the responses to three different neutron energies (3.15, 3.05 and 2.95 MeV, respectively).

**Figure 6:**
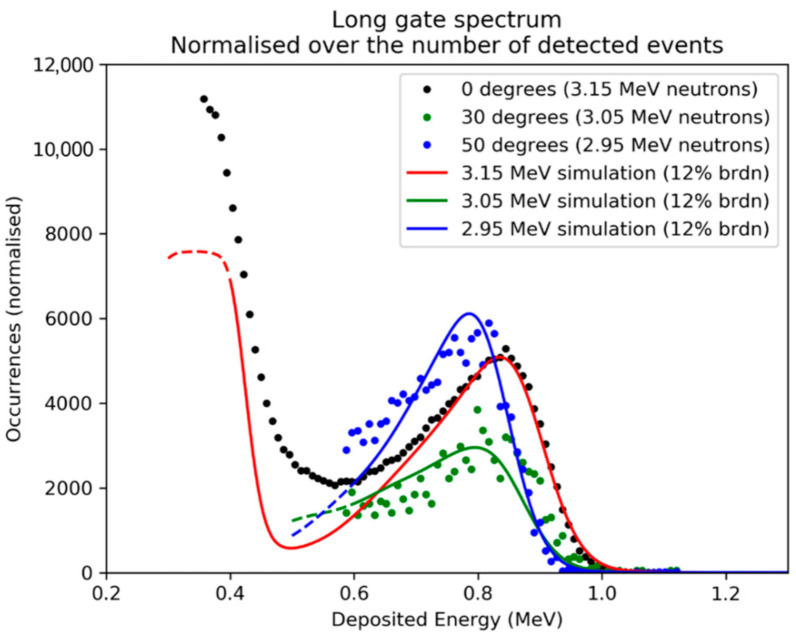
Deposited energy spectra for three different neutron energies at the FNG (3.1, 3.05 and 2.95 MeV). The number of events is normalized. The three sets are compared to three GEANT4 simulations, simulating $3\times 10^{8}$ monochromatic neutrons. The simulated spectra obtained were broadened through a Gaussian convolution with FWHM = 12% of the energy value, in order to emulate the detector’s energy resolution. Simulations are plotted as continuous lines and normalized to fit the data.

The pulse height spectrum obtained is shown in Figure 6, where the pulse height value was converted to $E_{d}$ by calibrating the detector with the $Am_{241}$ α-source used as described in Section 3.3. Energy thresholds of 300 keV for the measurement at 0° and 600 keV for the 30° and 50° measurements were used in order to ignore noise.

The spectra are dominated by the deposition of energy through elastic scattering reactions, whose cross section is dominant in the lower energy ranges ($E_{n}<4\;MeV$). Since in the scattering process, the energy that can be deposited ranges from 0 to a maximum value, the pulse height spectrum features an edge. The shoulder position depends on the neutron energy and on the target nucleus: elastic scattering on carbon $({}^{12}C\left(n,n\right){}^{12}C$) is clearly visible for $E_{d}≃0.8-0.9$ MeV for all three angles, while the edge due to scattering on silicon $({}^{28}Si\left(n,n\right){}^{28}Si$) is only visible for the 0° data at lower $E_{n}$. Table 2 shows that the $E_{d}$ of the four shoulders slightly overestimates the maximum $E_{d}$ based on theory, defined as a fraction of $E_{n}$ ($E_{d_{\max}}=0.28\times E_{n}$ for the carbon, $E_{d_{\max}}=0.13\times E_{n}$ for the silicon).

The measured spectra were then compared to the results of a Monte Carlo simulation performed with the GEANT4 code, in which the 3D geometry of the SiC active volume, the contacts and the aluminum front panel was reproduced. The interaction of $3\times 10^{8}$ neutrons from a 1-D monochromatic beam was simulated for each of the three energies. The simulated spectrum was then broadened with a Gaussian function, in order to simulate the effect of the detector’s energy resolution; the broadening chosen as a first approximation was 12% of the $E_{n}$ value. The simulated spectra obtained are plotted in Figure 6. The number of occurrences of the three angles was normalized to the simulation data.

## 6. Conclusions

The stability of two silicon carbide detectors (SiC) was tested under the irradiation of α-particles and neutrons. Both detectors were tested under a $≃10^{7}\frac{\alpha }{{\mathrm{mm}}^{2}}$ irradiation and proven to have stable responses, efficiencies and energy resolutions. Both detectors were also tested with fast spallation neutrons at two different ISIS facilities and proven to have stable response functions after $≃10^{9}\frac{n}{{\mathrm{mm}}^{2}}$ of neutron irradiation. This stability, which is orders of magnitude longer than the stability proven for diamonds in [17], makes the SiC a good candidate as a neutron counter or spectrometer for installation in the harshest environments, such as the breeding blankets of next-generation tokamaks. In order to achieve this, the functionality of the electronic chain, mainly, the preamplifier, will have to be tested in the future under similar levels of irradiation. An alternative could be to couple the SiC to a preamplifier capable of operating far away from the detector, as done for diamond detectors in [28].

Lastly, a SiC detector was tested with D-D quasi-monochromatic neutrons in order to complement the work in [14]. Three energies in the vicinity of $E_{n}$ = 3 MeV were tested, showing elastic scattering on carbon to be the most robust detection mechanism. The spectra were compared to an ideal simulation with a 12% energy resolution broadening, finding a good agreement. This confirms the possibility of using the SiC as a spectrometer for D-D neutrons.

## Figures and Tables

**Table 1:** Summary of the mean number of counts, pulse height and energy resolution measured during the alpha particle irradiation.

	**Mean No. of Counts, ** µ	**Dispersion (σ)**	**Poisson Uncertainty (** $=\sqrt{µ}$ **)**
SiC10	36,460.55	±195.02	190.95
SiC100	94,737.97	±313.11	307.80
	**Mean Pulse Height**	**Dispersion (σ)**	**Max PH—Min PH**
SiC10	7995.52	±8.09	-
SiC100	9672.03	±27.60	9709 − 9625 = 84 (0.87%)
	**Mean Energy Resolution**	**Dispersion (σ)**	
SiC10	4.356%	±0.113%	-
SiC100	2.893%	±0.036%	-

**Table 2:** Expected and measured positions of the elastic edges for both ^12^C and ^28^Si for the three positions.

	^12^C(n,n’)^12^C		^28^Si(n,n’)^28^Si	
Angle (deg)	Meas. (MeV)	Theor. (MeV)	Meas. (MeV)	Theor. (MeV)
0	0.91	0.882	0.43	0.4095
30	0.9	0.854	-	-
50	0.86	0.826	-	-

## Data Availability

The data presented in this study are available on request from the corresponding author. The data are not publicly available due to restriction imposed by the home institution.
